# Disrupting the SHOC2-RAS Nexus: a new frontier in targeting RAS-Driven cancers

**DOI:** 10.1186/s43556-025-00342-y

**Published:** 2025-11-27

**Authors:** Yudi Huang, Zhuoyang Chen, Shanqiang Qu

**Affiliations:** 1 Department of Neurosurgery, Nanfang Hospital, Southern Medical University, Guangzhou, Guangdong 510515 The People’s Republic of China; 2 The First Clinical School of Medicine, Southern Medical University, Guangzhou, Guangdong 510515 The People’s Republic of China; 3 Nanfang Glioma Center, Nanfang Hospital, Southern Medical University, Guangzhou, Guangdong 510515 The People’s Republic of China

In a recent *Nature* paper, Hauseman et al. identify the SHOC2–RAS interaction as a mutation-selective vulnerability in RAS(Q61*) tumors and develop Compound 6, a first-in-class inhibitor that disrupts this protein–protein interface [1]. This work establishes a new therapeutic paradigm for targeting RAS-driven cancers by disrupting critical co-factors, offering a promising strategy beyond direct RAS inhibition.

The RAS oncoproteins, frequently mutated in human cancers, have long been considered “undruggable” because of their picomolar affinity for GTP and the absence of deep pockets for small-molecule binding. While recent advances have yielded covalent inhibitors against KRAS(G12C), effective therapeutic approaches for other common RAS mutations—particularly those at the Q61 position—remain an unmet clinical need [2, 3]. However, Hauseman et al. shift the therapeutic paradigm from direct RAS targeting towards disrupting critical co-factors, opening a new avenue for a broad spectrum of RAS-driven cancers [1]. SHOC2 functions as an essential scaffold within the SHOC2–MRAS–PP1C (SMP) complex, which plays a pivotal role in RAF activation by dephosphorylating the autoinhibitory Ser259 site on RAF kinases. This dephosphorylation relieves RAF autoinhibition and permits stable association with GTP-bound RAS, thus sustaining MAPK signaling [4]. The SMP complex therefore acts as an essential molecular bridge, facilitating the transition of RAF from an inactive to an active state, and its integrity is crucial for full oncogenic RAS signaling output. This essential role of SHOC2 is underpinned by the foundational structural work of Liau et al., which defined the mechanism of SHOC2 modulation of RAS signaling [5]. Mechanistically, Q61R/L mutations in NRAS induce conformational rearrangements that remodel the switch I/II regions, thereby enhancing binding to the leucine-rich repeat (LRR) domain of SHOC2. Co-crystal structures revealed a distinct interface stabilized by salt bridges, hydrogen bonds, and hydrophobic contacts, providing a precise blueprint for structure-based drug design [1]. This structural insight is pivotal as it not only explains the mutation-specific dependency but also delineates a well-defined, druggable pocket on SHOC2, which is absent in its interaction with wild-type RAS (Fig. 1).

**Fig. 1:**
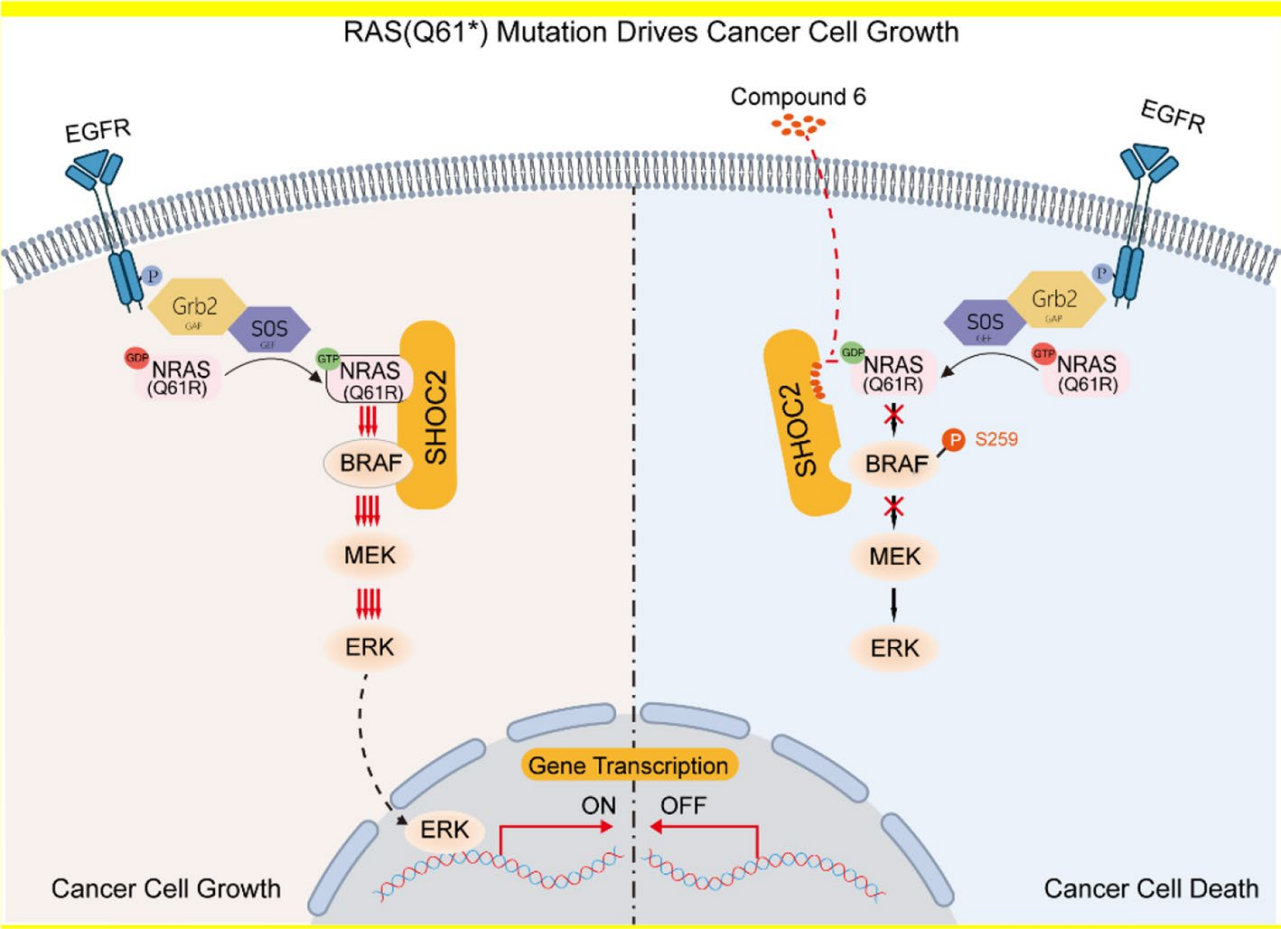
Targeting the SHOC2–RAS interaction inhibits oncogenic MAPK signaling. SHOC2 stabilizes the RAS-RAF complex, enabling RAF dephosphorylation and sustained MAPK pathway activation. Compound 6 blocks the interaction between SHOC2-NRAS(Q61R), leading to RAF hyperphosphorylation, ERK signaling suppression, and tumor cell apoptosis in RAS(Q61*)-mutant cancers. (This figure was created using Adobe Illustrator)

Guided by this structural blueprint, the authors performed high-throughput screening and structure-guided optimization to develop Compound 6, which occupies a defined pocket centered around R223/Q269 on SHOC2. The compound disrupts the SHOC2–RAS(Q61R) interaction with nanomolar potency (TR-FRET IC₅₀ ≈ 48 nM) and shows over 100-fold selectivity for Q61* mutants relative to wild-type RAS. This high degree of selectivity is a critical feature, as it minimizes the potential for on-target toxicity in healthy tissues that rely on basal SHOC2 function for normal MAPK signaling. In cellular models, Compound 6 disassembles the SMP complex, prevents RAF dephosphorylation at Ser259, and suppresses ERK activity, leading to selective cytotoxicity in KRAS/NRAS(Q61*) mutant cells while causing only transient ERK fluctuations in normal fibroblasts [1]. This phenomenon, known as synthetic lethality, wherein the inhibition of SHOC2 is selectively lethal only in the context of the RAS(Q61*) oncogene, underscores the potential for a wide therapeutic window. It is important to note that the substantial tumor regressions reported in the study were achieved through SHOC2 gene silencing rather than pharmacological inhibition. No in vivo efficacy or pharmacokinetic data for Compound 6 have yet been reported, underscoring its role as a proof-of-concept chemical probe rather than a therapeutic candidate. This distinction reflects a common trajectory in translational oncology, where genetic validation precedes the development of drug-like small molecules. The promising anti-tumor efficacy observed with genetic ablation strongly motivates the ongoing chemical optimization of Compound 6 and its analogues. Further optimization of membrane permeability, metabolic stability, and oral bioavailability will be required to convert this probe into a lead compound with in vivo utility. Key challenges include improving its solubility and plasma half-life to achieve sustained target coverage in vivo, which are essential prerequisites for evaluating efficacy in animal models.

Targeting an obligate cofactor such as SHOC2 offers both opportunities and challenges. Because SHOC2 acts upstream of RAF but downstream of RAS, disrupting the SHOC2–RAS interface could achieve mutation-selective MAPK pathway inhibition while reducing the adaptive feedback frequently observed with MEK inhibitors [3]. This upstream, mutation-selective intervention may preempt the pathway reactivation that often limits the efficacy of downstream inhibitors. However, SHOC2 also plays vital roles in normal physiology; germline mutations in components of the SHOC2–PP1C regulatory axis cause Noonan-like syndromes with cardiac abnormalities, necessitating careful on-target toxicity evaluation. Therefore, any future clinical development must include rigorous cardiac safety assessments. Comprehensive preclinical studies will therefore need to include PK/PD assessment, dose–toxicity relationships, and evaluation across multiple RAS(Q61) tumor models (e.g., melanoma, thyroid, and pancreatic cancers). Establishing predictive biomarkers, such as specific Q61 mutations and SHOC2 expression levels, will also be crucial for enriching patient populations most likely to respond to therapy. Future studies should also investigate mechanisms of resistance and rational combination strategies. Potential resistance routes may involve mutations within the SHOC2 binding pocket, reactivation of parallel signaling pathways, or reassembly of phosphatase complexes. To proactively address this, conducting long-term culture of RAS(Q61*) cells with escalating doses of SHOC2 inhibitors can help elucidate common resistance pathways. Combination therapies pairing SHOC2 inhibitors with MEK or ERK inhibitors could enhance efficacy and delay resistance, though overlapping toxicities require careful management. Alternatively, combinations with agents targeting cell cycle progression or apoptosis pathways (e.g., BCL-2 inhibitors) may yield synergistic effects without exacerbating MAPK pathway-related toxicities. In addition, exploring SHOC2 inhibition together with immunotherapy or metabolic modulators could further extend clinical benefit in RAS-mutant contexts. Beyond orthosteric blockade, emerging modalities such as allosteric inhibitors or proteolysis-targeting chimeras (PROTACs) that induce SHOC2 degradation may provide more durable target suppression and complement conventional small molecules. The catalytic nature of PROTACs could be particularly advantageous for achieving profound and sustained pathway suppression with intermittent dosing. In summary, the work by Hauseman et al. establishes a compelling structural and functional framework for targeting the SHOC2–RAS complex. Although Compound 6 is not yet a clinical candidate, it represents a transformative chemical probe that validates a new therapeutic paradigm—disrupting essential RAS cofactors to attenuate oncogenic signaling. This concept demonstrates that indirectly targeting RAS through its critical protein–protein interfaces is a viable and powerful strategy. The journey from this foundational discovery to a clinically effective therapeutic will be iterative, requiring close collaboration between structural biologists, medicinal chemists, and clinical oncologists. Further optimization, resistance mapping, and biomarker-guided studies will be key steps toward translating this discovery into effective treatments for patients with RAS(Q61*)-driven malignancies. This research illuminates a promising path forward in the relentless pursuit of conquering RAS-driven cancers, offering new hope for patients harboring these recalcitrant mutations.

## Data Availability

Not applicable.
